# Delirium and High Creatine Kinase and Myoglobin Levels Related to Synthetic Cannabinoid Withdrawal

**DOI:** 10.1155/2017/3894749

**Published:** 2017-04-10

**Authors:** Ahmet Bulent Yazici, Esra Yazici, Atila Erol

**Affiliations:** ^1^Department of Psychiatry, Training and Research Hospital, Sakarya University, 54100 Sakarya, Turkey; ^2^Department of Psychiatry, Faculty of Medicine, Sakarya University, 54100 Sakarya, Turkey

## Abstract

Synthetic cannabinoids (SCs) are included in a group of drugs called new psychoactive substances. Effects of SCs on the central nervous system are similar to other cannabinoids, but 2–100 times more potent than marijuana. Thus, addiction and withdrawal symptoms are more severe than natural cannabinoids. Withdrawal symptoms of SCs were reported in the literature previously. But there is no report about SC withdrawal delirium and its treatment. Several studies reported that agonists of CB1 receptors play a role in GABA and glutamatergic neurotransmission, which is similar to the effects of alcohol on GABA and glutamatergic receptors. Previous studies on alcohol delirium cases suggested that elevated creatine kinase (CK) can be a marker of progress. This study reports delirium and high serum CK levels related to SC withdrawal and offers a treatment with benzodiazepine for them. We described two cases treated in our inpatient clinic about SC withdrawal with increase of serum CK level and other laboratory parameters. One of them demonstrated delirium symptoms and the other did not with early rapid treatment.

## 1. Introduction

Cannabis use disorder is defined as “the problematic pattern of cannabis use leading to clinically significant impairment or distress, as manifested by at least two symptoms, forming within a 12-month period” by DSM-V (The Diagnostic and Statistical Manual of Mental Disorders). Cannabis use disorder and the other cannabis-related disorders contain problems which are associated with substances derived from the cannabis plant and chemically similar synthetic compounds [1].

Cannabis can be classified as endocannabinoids, phytocannabinoids, and synthetic cannabinoids [2]. Synthetic cannabinoids (SCs) are included in a group of drugs called new psychoactive substances [3, 4]. SCs were synthesized in clandestine laboratories and they were sprayed on dried various plant materials. SCs were first marketed as natural cannabis alternatives in Europe in 2004, USA in 2008, and Turkey 2010. SCs are widely used in Turkey and around the world in recent years. They have hundreds of chemical types and these are usually called K2, Spice, Bonzai, Jamaika, and so forth [1] It has both similar and different pharmacokinetic and pharmacodynamic mechanisms with major active component of marijuana (Δ(9)-tetrahydrocannabinol-THC) [5, 6].

Effects of SCs on the central nervous system are similar to other cannabinoids [7, 8]. Considering these effects, cannabinoid ligands bind with specific G protein-coupled receptors (CB1 receptor and the CB2 receptor) and activate the endocannabinoid system.

SCs have potent agonism to cannabinoid receptors but THC has partial agonism [6]. CB1 receptors influence the central nervous system and cause thermoregulation disorders, psychotic episodes, memory disorders antiemetic activity, appetite enhancer activity anxiety, and stress relieving activities [9, 10]. SCs were shown to have 2–100 times more potent pharmacological effects and they have greater physiological and psychoactive effects than THC [11].

DSM-V describe cannabis-related withdrawal symptoms but different symptoms for SC were not described in DSM-V. There are a growing number of reports about SC withdrawal symptoms. It seems that SC withdrawal symptoms are more severe than THC related withdrawal symptoms. SC withdrawal was reported at the daily-regular users when they abrupt to use it [12]. Onset of withdrawal symptoms reported changing between 2 hours to one week after last smoking. Severity of symptoms seems to correspond to amount of daily SC use. These symptoms include agitation, irritability, mood swing, vivid dreams, seizures, tachycardia, tremor, chest pain, cramping palpitations, dyspnea, cravings, headache, severe anxiety, insomnia, nausea and vomiting, loss of appetite, and diaphoresis [12]. According to majority of case reports withdrawal symptoms were treated by benzodiazepine and quetiapine. But there is no report about SC withdrawal delirium and its treatment. This study reports delirium and high serum creatine kinase (CK) and myoglobin (MB) levels related to SC withdrawal and offers a treatment for them.

We described two cases treated in our inpatient clinic (Sakarya Training and Research Hospital (STRH)) about SC withdrawal with increase of serum CK level and other laboratory parameters. One of them demonstrated delirium symptoms and the other did not with early rapid treatment.

STRH inpatient clinic houses had 21 beds for women, 21 beds for men, and 12 beds for substance users. In the SRTH psychiatry and Alcohol and Substance Addiction Treatment and Education Centre (AMATEM) wards were routinely screened by hemogram, serum thyroid function tests, B12 level, folic acid level, D vitamin level, hepatitis A-B-C, HIV markers, other serum biochemical parameters, and urine drug analysis (Tables 1(a) and 1(b)).

To evaluate of delirium level of Case 1 we used New Delirium Rating Scale (NDRS). Development, reliability, and validity of NDRS were conducted by Ok et al. [13]. The NDRS is a 10-item clinician-rated scale to assess delirium severity with each item rated on a Likert scale (0–3) and cut of point was accepted as 11.

## 2. Case 1

A 26-year-old single male patient who had been graduated from secondary school and had no regular job was admitted to our hospital. Accompanied by his family, the patient voluntarily presented to AMATEM outpatient clinic. On presentation, he complained that he was nervous and short-tempered after he quit using the substance called “bonzai” 2 days ago; he did not sleep at night and became aggressive. His family said that he became very active and talked constantly after quitting the substance 2 days ago; he did not sleep and became aggressive when he was prevented or when his wishes were not met. According to his history, he began to smoke marijuana when he was 12, followed by ecstasy and crack cocaine use several times a month; at the age of 16, he was hospitalized in an addiction clinic, received treatment, and stopped using substance for some time. After about 4 years, he began to use marijuana and ecstasy again with the influence of his friends. He was caught using substance 4 years ago and began to use SCs to prevent detection of drugs in urine drug test, while he was on probation. 6 months ago, he was hospitalized in STRH AMATEM clinic and voluntarily received treatment for 4-5 days, and his serum CK, MB values were normal in tests performed during his hospitalization. He completely stopped using substance after discharge but resumed using SC intensively about 1 week ago. He used crack cocaine once in that period. 2 days ago; he voluntarily stopped using substance completely and presented to our AMATEM outpatient clinic with the existing complaints with the intention of quitting substance. In his psychiatric examination, manic symptoms were prevailing so he was referred to psychiatric ward and admitted for tests and treatment.

In his mental status examination (first examination), he was alert, fully oriented, and cooperative; he was gentle to the interviewer and his attitude was cooperative; his hygiene and grooming were adequate; his mood was elevated; his affect was euphoric and he was irritable; his distractibility and speech content were increased; he was elaborative; his associations were fast; thoughts and attitudes were mild grandiose, but no delusions in thought content or pathology in perception were identified. Psychomotor activity was increased. His insight and judgment were inadequate.

In clinical course and treatment, when he was admitted to the clinic, it was observed that he had expansive mood and irritability, paranoid tendencies (jealousy thoughts related to his girlfriend), and elementary auditory hallucinations (being called out by his name). Upon an increase in his excitation, haloperidol 10 mg and biperiden HCl 2 mg IM were administered, and quetiapine 500 mg/day and risperidone 2 mg/day were added to his treatment. He said he had craving and discomfort so he was given diazepam 5 mg (Table 2).

Other than elevated CK, his hematological and biochemical values were within normal limits. Substance was not detected in urine substance test (Table 2). Despite being restless and irritable in the clinic, he was responsive to suggestions and cooperative. In the next 3 days, he was administered quetiapine 3 × 100 and risperidone 3 mg. He was generally calm and observant of clinical rules.

*Beginning of Delirium*. In the evening of day 4 of hospitalization, he began to show the following: deterioration of place and time orientation, auditory-visual hallucinations (self-conversation, murmurs, and trying to catch something in the air), delusions (thinking that people around are being changed and saying that he is trapped in a game and nurses are devils and are trying to harm him), disorganized behaviour (random spitting and wandering about), singing songs, trying to get rid of mechanical stabilization, nonsense laughs, and not sleeping. Delirium evaluation scale was applied to the patient as from day 4.

His tests were repeated and his lab results revealed seriously elevated CK and CK-MB values (Table 2, days 3-4). His blood pressure and pulse were normal. Assessment of rigidity and other extrapyramidal system symptoms revealed no finding. Troponin and INR PTZ values requested as a result of consultation of internal diseases department were normal. His treatment was continued with 2000 cc of 5% dextrose and lorezepam mg 2 × 1/2. Despite treatment, his symptoms became severe so he was transferred to AMATEM interim intensive care unit. In his tests were performed on his 1st day in AMATEM, CK: 4267, which was higher than the measurable values, and in addition, CK-MB and MB values were elevated (Table 3, day 5).

His vitals were normal, except mild tachycardia and mild hypertension (max 150/100). His delirium symptoms increasingly continued and lorezepam 9.5 mg/g sublingual, 3000 cc of fluids (1500 isotonic + 1500 5% dextrose), and haloperidol 1 × 5 mg IV were given to the patient. He also developed urinary incontinence. Although there was a mild and temporary improvement in the excitation picture with the existing treatment, the patient was disoriented except for short periods, and his excitation picture continued so in order to provide a complete sedation, he was taken to general ICU (intensive care unit) and continued to be followed. The patient's treatment was continued in intensive care unit with 2.5 mg lorezepam SL 30 mg/g diazepam IV, 3000 cc/g of IV fluids, and 10 mg/g haloperidol IV. The patient calmed down with this treatment and his psychomotor activity and agitation excitation abated. His repeated substance test result was negative for substance. His mental fog and auditory-visual hallucinations continued for another two days. His CK, CK-MB, and MB values began to decrease (Table 2, days 7 and 8). He completely recovered on day 8 of hospitalization and day 3 of his follow-up at ICU. As soon as he regained consciousness, he stated that he wanted to be discharged. He was followed up at outpatient clinic.

It was learned that he began to use SC again 2 days after his discharge. 3 months later, he presented with the intention of quitting SC and had mild hypomanic symptoms at the time. The frequency and quantity of SC use were less than before. He was started on 200 mg carbamazepine, 100 quetiapine, 5 mg olanzapine, and 10 mg diazepam. CK values were normal at baseline and follow-up. No additional clinical pathology suggesting delirium and restlessness, tension, and so forth was observed. He was discharged on day 6 of hospitalization upon his request.

## 3. Case 2

A 30-year-old male, born in Izmit, a high-school graduate, driver, married, was included.

The patient was urgently referred from a psychiatric clinic and voluntarily presented to AMATEM polyclinic accompanied by his father.

His complaint on presentation was “I was using bonzai. I have not used it for 2 days but I drank alcohol but I did not feel relaxed, I want to be admitted to the clinic.” According to the information received from his family, he had not been using substance for 2 days; he was very nervous and did not sleep. The biochemical analysis of the patient revealed that the laboratory levels were high (CK: 1233) so he was admitted for tests and treatment.

According to the story taken from him and his family, he began to smoke marijuana at the age of 12, used drugs including methamphetamine, MDMA, and heroin, and he was jailed for 3 years between the ages of 23 and 26 due to substance use (2009–2012) so he was in mandatory remission but he started to use substance again as soon as he was discharged. He continued to use SC and marijuana on and off for the last 5 years; he has been using SC and marijuana almost every day for the last 1 month but voluntarily stopped using SC and marijuana 2 days ago. After he quit SC, he suffered from tension, contractions, nervousness, nausea, and vomiting and he drank 13 standard drinks once to relieve his complaints, which did not fully abate, and diazepam 10 mg was given intramuscularly twice to the patient at emergency department.

In his mental status examination, he was alert, fully oriented, and cooperative; he was open to communication and partly cooperative with the interview. He made eye contact; memory and intelligence were normal; he was irritable, anxious, and occasionally angry; he had reduced attention and concentration and normal flow of thoughts and connotations; no pathology in thought content and perception; reasoning and insight were preserved; insomnia and psychomotor unrest were present.

*Clinical Observation and Treatment*. On day 1 of his hospitalization, CK: 1233, CK-MB: 93.1, AST (aspartate aminotransferase) 39, MB: 413, and his substance test revealed THC: 88 nanogram/mL, positive benzodiazepine and ethyl glucuronide (Table 3). Parenteral benzodiazepine (10 mg diazepam in 1500 cc of isotonic), 100 mg quetiapine, and mirtazapine 30 mg were ordered. Sleep was not achieved with these medications and the restlessness of the patient decreased after diazepam administration but increased in general.

On day 2, he had agitation and insomnia with fluctuations in irritability and emotion, and his repeated biochemistry tests revealed CK: 2954, CK-MB: 230, and AST: 74. The patient's treatment was continued with 1500 cc/g isotonic 30 mg/g diazepam IV and 5 mg/g haloperidol IV and 2.5 mg/g lorezepam. After the treatment, the patient's irritability was reduced, and his sleep was regulated. He was alert, cooperative, and oriented. His repeated substance test revealed THC 91 nanogram/mL and the patient tested positive for benzodiazepine and negative for ETG (Table 3). On days 3, 4, 5, 6, and 7, treatment was continued in exactly the same way. His biochemical analyses showed normal results and the patient was observed to be calm and compliant (Table 3). On day 8, oral diazepam 10 mg and lorezepam 2.5 mg were administered. 500 cc isotonic was given. He denied any complaints and was voluntarily discharged.

## 4. Discussion

In this paper, two cases that presented with SC use and showed different courses are described. The common features of these two cases were elevated CK levels immediately after quitting SC and good response to benzodiazepine during detoxification.

Elevated CK and AST levels of these cases can be explained in two different ways. The first explanation that comes to mind is a picture that develops due to substance intoxication. Elevations in CK, CK_MB, and AST parameters due to SC intoxication were previously reported in case reports [14, 15]. However, a systematic review on 3965 cases reported rhabdomyolysis in only 0.1% and delirium in 0.2% of SCs toxication cases [16]. However, interestingly, in both cases presented in this paper, substance intoxication symptoms did not start while the patient was using substance or immediately after he quit it but rather at least 2 days after he quit substance, and in one case, the clinical findings and biochemical parameters reached peak levels on day 6 of hospitalization and significantly decreased on day 8 (Table 2). In the second case, biochemical parameters reached peak level for 2 days but the patient did not develop delirium (Table 3). Neither patient tested positive in urine substance test. It is noteworthy that in the first case, the patient used SC quite intensely before deprivation period during which delirium was observed and that he did not develop delirium in the other periods when the deprivation began. In the second case, the patient drank alcohol for self-medication and was given diazepam in emergency department, which might have prevented worsening of picture in early intervention period and development of delirium.

SCs are central depressants in effect, and fluid support, medical care, and ensuring fluid-electrolyte balance are recommended in the case of development of intoxication. Benzodiazepines are recommended for agitation, irritability, anxiety, and seizures in the case of both deprivation and intoxication [12]. There are many SCs, some of which are long-acting, and some are short-acting, and their potencies are quite different [11]. In general, it is assumed that the effects of SCs taken by inhalation start within minutes-hours and end before 8 hours. This may be slightly longer in oral intake [17]. The patients in this study used SC by inhalation. The likelihood of redistribution, such as THC stored in fat tissue, may also come to mind [18]. The only evidence that can be assessed in this respect is that SC values of the first patient increased but remained below the threshold in his repeated substance test. He did not have any previously described SC deprivation delirium picture, but deprivation has been described in several case reports [11, 12]. In this paper, we think that SC deprivation can lead to delirium like alcohol, and we claim that early benzodiazepine is important to prevent this situation. Indeed, previous studies on alcohol delirium cases suggested that elevated CK can be a marker, which was the case for two patients in the current study [19]. Several studies reported that agonists of CB1 receptors play a role in GABA and glutamatergic neurotransmission, which is similar to the effects of alcohol on GABA and glutamatergic receptors [20–22]. The effects of cannabinoids on autonomic neurons have been reported [23] and cannabinoid deprivation reduces autonomic hyperactivity and resembles alcohol deprivation in this respect. In many case reports, benzodiazepines have been reported to be useful in SC deprivation [12]. The neuroprotective effect of CK in the nigrostriatal pathway has been mentioned, and it is claimed that elevated CK during delirium is related to this effect [19]. In the light of this information, in the current two cases, there appears to be evidence that there's a relationship between elevated CK and the severity of SC deprivation and that early benzodiazepine administration can prevent delirium development, as in the case of alcohol deprivation.

However, the most important problem with this paper seems to be the low reliability of information provided by the substance users, the high number of SCs and their different efficacies, and the lack of knowledge about their concentration in the under-the-counter products. So this makes it impossible to know the type and amount of the SC used by the person who presented with SC use and in which mixture s/he uses it. This in turn makes it difficult to make a complete and accurate interpretation. Despite that, there is a theoretically possible and logical interpretation in these two cases. Apparently, there is a need for further studies on the detection of SCs, their pharmacokinetics and pharmacodynamics, and their toxic and deprivation effects.

## 5. Conclusion

In the use of SCs, presentation with elevated CK values may be related to delirium. The use of benzodiazepine in treatment is useful as both inhibitor and a therapeutic option.

## Figures and Tables

**Table:** (a) Urine drug tests and cut-off levels

Urine drug tests	Cut-off levels
Delta-9-tetrahydrocannabinol (THC)	50 ng/mL
Cocaine	300 ng/mL
Amphetamine	500 ng/mL
3,4-Methylenedioxymethamphetamine (MDMA)	500 ng/mL
Ethyl glucuronide (ETG)	500 ng/mL
Benzodiazepine	300 ng/mL
6-Acetyl morphine	10 ng/mL
Opiate	300 ng/mL
JWH-18(synthetic cannabinoid-SC-1)	20 ng/mL
JWH-073(SC-1)	20 ng/mL
AM-2201(SC-1)	20 ng/mL
UR-144(SC-2)	10 ng/mL
XLR-11-N-4 derivatives(SC-2)	10 ng/mL
Barbiturates	300 ng/mL
Buprenorphine	5 ng/mL

**Table:** (b) Normal ranges of biochemical parameters

Myoglobin (MB)	0–154,9 ng/mL
Serum creatine kinase (CK)	30–200 U/L
Serum creatine kinase MB (CK-MB)	0–25 U/L
Serum aspartate transaminase	0–34 ng/mL

**Table 2:** Case 1 clinical observation and treatment.

Day	Psychiatric evaluation	Biochemical parameters						Treatment
		CK^1^	CK-MB^2^	MB^3^	AST^4^	SC-1^5^	SC-2^6^	
1	Manic symptoms (euphoria, irritable mood, logorrhoea, delusion)	796	48,4		39	1.5	2,3	Haloperidol 10 mg/d, quetiapine 400 mg/d, risperidone 2 mg/d, diazepam 5 mg/d

2	Manic symptoms							Quetiapine 300 mg/d, risperidone 2 mg/d

3	Manic symptoms							Quetiapine 300 mg/d

4	Delirium symptoms NDRS:15	2571	66,4		67			5% dextrose 2000 cc/d, risperidone 2 mg/d, lorazepam 2,5 mg/d

5	Delirium symptoms NDRS:29	4267 ↑	220	708	166	7,22	8,08	Lorazepam 9,5 mg/d, 5% dextrose 1500 cc/d, 0,9% isotonic 1500 cc/d Haloperidol 10 mg/d

5	Delirium symptoms NDRS:30	4267 ↑	130	564	140			Lorazepam 2,5 mg/d, diazepam 30 mg/d 5% dextrose 1500 cc/d, 0,9% isotonic 1500 cc/d

7	Delirium symptoms NDRS:12	2049	56,9	262	96			Diazepam 20 mg/d 5% dextrose 1500 cc/d, 0,9% isotonic 1500 cc/d Haloperidol 10 mg/d

8	Hyperthymia NDRS: 3	1429	43,7	157	77			Diazepam 20 mg/d 0,9% isotonic 500 cc/d

^1^Creatine kinase, ^2^creatine kinase-MB, ^3^myoglobin, ^4^aspartate aminotransferase, ^5^synthetic cannabinoid-1 (Table 1), and ^6^synthetic cannabinoid-2 (Table 1); ↑ test results exceed the measurable values.

**Table 3:** Case 2 clinical observation and treatment.

Day	Psychiatric evaluation	Biochemical parameters							Treatment
		CK^1^	CK-MB^2^	MB^3^	AST^4^	THC^5^	BNZ^6^	ETG^7^	
1.	Insomnia Irritability Agitation	1233	93,1	413	39	88	0	2000↑	0,9% isotonic 1500 cc/d Diazepam 10 mg/d, quetiapine 100 mg/d Mirtazapine 30 mg/d

2.	Insomnia Irritability Agitation Instable mood	2954	230		74	91	3000↑	94	0,9% isotonic 1500 cc/d Diazepam 30 mg/d Lorazepam 2,5 mg/d, haloperidol 5 mg/d

3.	Stable mood Normal sleep and behavior	1840	28		57				0,9% isotonic 1500 cc/d Diazepam 30 mg/d Lorazepam 2,5 mg/d, haloperidol 5 mg/d

4.	Stable mood Normal sleep and behavior	1408	25		50				0,9% isotonic 1500 cc/d Diazepam 30 mg/d Lorazepam 2,5 mg/d, haloperidol 5 mg/d

5.	Stable mood Normal sleep and behavior	1042	19,8		49	68,6	3000↑	48	0,9% isotonic 1500 cc/d Diazepam 30 mg/d Lorazepam 2,5 mg/d, haloperidol 5 mg/d

6.	Stable mood Normal sleep and behavior	493	14,2		40				0,9% isotonic 1500 cc/d Diazepam 30 mg/d Lorazepam 2,5 mg/d, haloperidol 5 mg/d

7.	Stable mood Normal sleep and behavior	234	13,7		35,4				0,9% isotonic 1500 cc/d Diazepam 30 mg/d Lorazepam 2,5 mg/d, haloperidol 5 mg/d

8.	Stable mood Normal sleep and behavior								Diazepam 10 mg/d, 0,9% isotonic 500 cc/d Lorazepam 2,5 mg/d

^1^Creatine kinase, ^2^creatine kinase-MB, ^3^myoglobin, ^4^aspartate aminotransferase, ^5^Δ(9)-tetrahydrocannabinol, ^6^benzodiazepine, and ^7^ethyl glucuronide; ↑ test results exceed the measurable values.
